# Matrikines dictate the amplitude of inflammation in smoke-related *Streptococcus pneumoniae* pulmonary infection

**DOI:** 10.1172/jci.insight.203605

**Published:** 2026-07-14

**Authors:** Sarah W. Robison, Jindong Li, Kristopher R. Genschmer, Liliana Viera, Jeremy B. Foote, Landon Wilson, W. Edward Swords, J. Edwin Blalock, Amit Gaggar, Xin Xu

**Affiliations:** 1 School of Medicine and Lung Biology Program, The University of Alabama at Birmingham, Birmingham, Alabama, USA.; 2 Medicine Service, Birmingham VA Medical Center, Birmingham, Alabama, USA.

**Keywords:** Inflammation, Pulmonology, Neutrophils, Proteases

## Abstract

Smoking remains a major risk factor for the severity of bacterial pneumonias, although mechanisms driving this injury remain poorly understood. This manuscript provides a new insight on how the smoke-exposed lung and bacteria augment the release of enzymes capable of directly damaging pulmonary tissue, leading to worsening inflammation and tissue damage.

**To the Editor:** Exposure to cigarette smoke (CS) increases susceptibility to respiratory infections. Among respiratory pathogens, *Streptococcus pneumoniae* (*SP*) remains a leading cause of community-acquired bacterial pneumonia globally, accounting for significant morbidity and mortality (1). Despite the mechanisms underlying enhanced lung injury and sustained neutrophilic inflammation remaining poorly understood, recent studies have highlighted that matrikines, such as proline-glycine-proline (PGP), are derived from collagen and play an important role in inflammation and tissue remodeling in lung disorders (2). PGP peptides act on CXCR1 and CXCR2 receptors on neutrophils, inducing chemotaxis and neutrophil activation (2). We hypothesized that combined exposure to CS and *SP* infection amplifies lung immune responses, leading to heightened inflammation via generation of PGP.

To test our hypothesis, we employed a 2-hit murine model of CS exposure and pneumococcal infection over a 2-week period (See Supplemental Methods; supplemental material available online with this article; https://doi.org/10.1172/jci.insight.203605DS1). To neutralize PGP, mice were administrated the PGP-specific peptide inhibitor L-arginine-threonine-arginine (RTR) intranasally and intratracheally 1 hour before smoke (3). Mice were analyzed using nasal wash, bronchoalveolar lavage fluid (BAL), and lung tissue.

We first investigated the effect of CS extract (CSE) and *SP* on prolyl endopeptidase (PE) and MMP-9 (key enzymes involved in PGP generation) (2) release from neutrophils. PE activity and MMP-9 levels were markedly elevated in the CSE and *SP*-exposed group (Figure 1, A and B). To investigate whether *SP* infection amplifies PGP expression and neutrophilic inflammation in CS-exposed mice, we administered *SP* intranasally to mice after 2 weeks of CS exposure. Mice exposed to CS and *SP* exhibited significantly greater body weight loss (Supplemental Figure 1A) and higher pulmonary bacterial burden (Figure 1C) compared with mice exposed to *SP* alone. Neutrophil counts and MPO were elevated (Figure 1, D and E), and lung histopathology revealed neutrophilic and mononuclear inflammation around vessels and extending into the alveolar interstitial space (Supplemental Figure 1, B and C). IgM levels increased in BAL (Figure 1F). PE activity (Figure 1G), MMP-9 (Figure 1H), and PGP levels (Figure 1I) were all elevated in CS and *SP*-exposed mice. These data demonstrate that CS and bacterial infection enhance PGP production and neutrophilic inflammation.

To evaluate the effects of PGP inhibition on neutrophilic inflammation in the 2-hit model, mice were pretreated with RTR and analyzed for inflammatory markers (4). PGP levels were reduced in CS- and *SP*-exposed mice treated with RTR (58.4% ± 13.4% reduction), confirming effective neutralization. RTR mitigated weight loss (8.6% weight loss for CS+*SP* vs. 4.6% for CS+*SP*+RTR, *P* < 0.05 by Mann-Whitney) and reduced neutrophil accumulation in nasal wash (Figure 1J). MPO was significantly reduced in BAL (Figure 1K), lung (Figure 1L), and nasal wash (Figure 1M). MMP-9 levels were also decreased in lung (Figure 1N) and nasal wash (Figure 1O). These data indicate that PGP inhibition attenuates airway neutrophilic inflammation after smoke and bacteria challenge.

In conclusion, this study provides mechanistic insight into how cigarette smoke primes the lung for exaggerated pneumococcal inflammation via a PGP-dependent pathway. We identify the MMP-9/PE-PGP axis as a key driver of smoke-associated pneumococcal pneumonia (Figure 1P). A major translational observation is that PGP inhibition mitigated inflammation and injury in CS- and *SP-*exposed mice, consistent with prior studies where RTR neutralized PGP-driven neutrophilia (4). Notably, unlike our previous study of chronic smoke-induced injury, this work demonstrate that the PGP axis is activated during acute pneumococcal infection in smoke-primed lung, highlighting a broader role for PGP in infection-associated inflammation. Therefore, PGP may serve both as a biomarker and a therapeutic target, opening new avenues for adjunctive treatment strategies in patients with pneumonia who smoke (5).

Our work extends the concept that matrikines regulate the amplitude and persistence of lung inflammation during infection, but it has limitations. The model used short-term CS exposure and a single pneumococcal challenge; longer exposures, different serotypes, and comorbidities (e.g., viral coinfection) may alter protease activation and PGP kinetics. In addition, we only tested RTR as a preexposure to both smoke and *SP*, and further studies should be conducted to evaluate RTR either after smoke and/or after *SP* exposure. Furthermore, the ex vivo neutrophil experiments could made further translational with complementary experiments done on human neutrophils. Finally, future studies should examine utilizing an unbiased, multi-omics approach to more broadly identify additional mechanisms operative in immune priming within the smoked and infected lung.

## Funding support

This work is the result of NIH funding, in whole or in part, and is subject to the NIH Public Access Policy. Through acceptance of this federal funding, the NIH has been given a right to make the work publicly available in PubMed Central.

- NIH grants R35HL135710 (to JEB) and R01HL162705 (to KRG).
- Veterans Administration Merit Review 101CX002751 (to AG) and VISN 7 Research Development Award (to SWR).

## Conflict of interest

The authors have declared that no conflict of interest exists.

## Supplementary Material

Supplemental data

Supporting data values

## Figures and Tables

**Figure 1:**
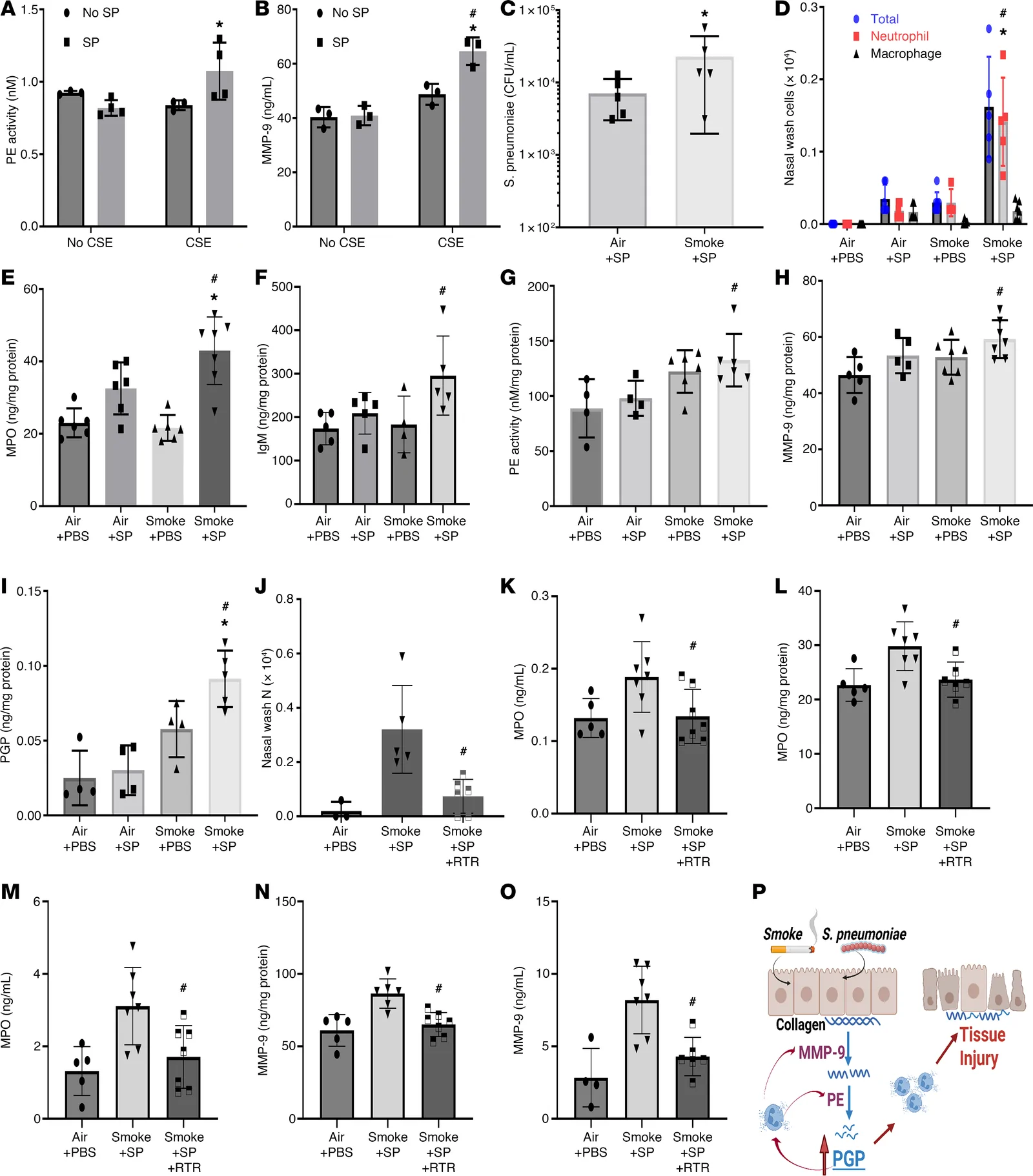
Matrikine PGP is involved in *S*. *pneumoniae* and cigarette smoke–induced respiratory inflammation. (**A** and **B**) Bone marrow–derived neutrophils from C57BL/6 mice were stimulated with cigarette smoke (CS) extract (CSE, 0.1%) and 2 × 10^4^ CPU/ml *S*. *pneumoniae* (SP) (EF3030, a serotype 19F). PE activity and MMP-9 ELISA assay were performed on the supernatants after stimulation for different times as indicated: (**A**) PE (30 minutes); (**B**) MMP-9 (1 hour). *P < 0.001 vs. No CSE+SP; ^#^*P* < 0.05 vs. CSE+No SP (2-way ANOVA with Tukey’s multiple comparison post test). (**C**–**I**) Mice were exposed to CS for 2 weeks, and 2 × 10^6^ CPU *SP* was administered intranasally 2 days before sample collection. (**C**) Bacteria count in the lung (*P < 0.05 vs. Air+SP, Mann-Whitney test). (**D**) Nasal wash cell count. (**E**) MPO in the lung lysate and (**F**) IgM in the BAL, as determined by ELISA. (**G**) PE activity in the lung lysate. (**H**) MMP-9 in the lung lysate. (**I**) PGP in the lung lysate as determined by electrospray ionization–liquid chromatography/tandem mass spectrometry (ESI-LC/MS/MS). ^#^*P* < 0.05 vs. Air+PBS. *P < 0.05 vs. Air+*SP* (1-way ANOVA with Tukey’s multiple comparison post test). (**J**–**O**) PGP-specific peptide inhibitor RTR was administered to mice intratracheally and intranasally 5 days a week over 2 weeks before they were exposed to CS. Samples were collected 48 hours after intranasal *SP* challenge. (**J**) Nasal wash neutrophils. (**K**) BAL MPO. (**L**) Lung lysate MPO. (**M**) Nasal wash MPO. (**N**) Lung lysate MMP-9. (**O**) Nasal wash MMP-9. ^#^*P* < 0.05 vs. Smoke+SP (Mann-Whitney test). All values represent mean ± SD. (**P**) Schema of proposed mechanism [BioRender. Xu, X. (2026) https://BioRender.com/qdish2c].

## Version 1 07/14/2026

In-Press Preview

## Version 2 08/24/2026

Electronic publication
