# Supplementary material for: Matrikines dictate the amplitude of inflammation in smoke-related Streptococcus pneumoniae pulmonary infection
Source: JCI Insight. 2026 Jul 14;11(16):e203605. doi: 10.1172/jci.insight.203605 (PMC13502163; doi:10.1172/jci.insight.203605)
Supplement: Supplementary file 1 [file jciinsight-11-203605-s196.pdf]

**Matrikines dictate the amplitude of inflammation in smoke-related Streptococcus pneumoniae pulmonary infection**

Sarah W. Robison[1,2], Jindong Li[1,2], Kristopher R. Genschmer[1], Liliana Viera[1], Jeremy B. Foote[1], Landon Wilson[1], W Edward Swords[1], J. Edwin Blalock[1], Amit Gaggar[1,2]*, Xin Xu[1,2]*#

[1]School of Medicine and Lung Biology Program, The University of Alabama at Birmingham, Birmingham, AL 35294, USA

[2]Medicine Service, Birmingham VA Medical Center, Birmingham, AL 35233, USA

*These authors contributed equally to this work

#Corresponding author: 845 19th St. S., Birmingham, AL 35294; Tel. 205-975-5015; xinx@uab.edu

## Supplemental Materials and Methods

### *Sex as a biological variable*

Only female C57BL/6 mice (6–8 weeks of age, purchased from Jackson Labs) were included in this study. Female mice were selected to provide experimental consistency and because the primary objective was to define the contribution of the MMP-9/PE–PGP pathway to smoke-associated pneumococcal inflammation rather than to compare sex-specific responses. Although previous studies have demonstrated sex-related differences in susceptibility to *Streptococcus pneumoniae* infection (1), there is no evidence that the protease–matrikine pathway investigated in the present study is restricted to one sex. Therefore, the findings are expected to be broadly relevant to both male and female hosts. Future studies examining sex as an experimental variable will be necessary to determine whether the magnitude of PGP generation or the response to RTR differs between sexes.

***Streptococcus pneumoniae***

*Streptococcus pneumoniae* (serotype 19, strain E3030) was cultured at 37 °C in 5% $CO_2$ on blood agar plates or in Todd-Hewitt broth supplemented with 0.5% yeast extract (THY broth) to an $OD_{600}$ of ~0.6-0.7 (~$1 \times 10^8$ CFUs/ml) and stored at −80 °C in 20% glycerol as single-use aliquots.

***Cigarette smoke extract and bacteria treatment of neutrophils***

Cigarette smoke extract (CSE) was freshly prepared by passing smoke from 3R4F research cigarettes (Kentucky Tobacco Research and Development Center) through 10 mL of pre-warmed (37 °C) 0.1% BSA/HBSS using a custom smoking apparatus; concentration was standardized at 320 nm. Bone marrow neutrophils were isolated from female C57BL/6 mice via Histopaque gradient, resuspended in HBSS/0.1% BSA ($2 \times 10^6$ cells/mL; purity >95%, viability >98%), and plated in 96-well plates at 0.1 mL/well ($2 \times 10^5$ cells). Cells were treated with 4% CSE for 1 h, followed by *S. pneumoniae* ($2 \times 10^5$ CFUs) for 30 minutes (PE levels) or 1 h (MMP-9 levels) at 37 °C, 5% $CO_2$. Supernatants were collected and stored at −80 °C for subsequent PE and MMP-9 assays.

***Two-hit murine model combining S. pneumoniae infection with cigarette smoke exposure***

Mice (6-8 week old C57BL/6, female) were exposed to smoke generated by a SCIREQ "InExpose" smoking system (SCIREQ, Canada) using 3R4F reference cigarettes for two weeks. Those mice challenged with $2x10^6$ CFUs *S. pneumoniae* were inoculated intranasally at two days before sample collection. To neutralize PGP, mice were intratracheally and intranasally administrated the PGP-specific peptide inhibitor L-arginine-threonine-arginine (RTR) at a dose of 250 μg one hour before smoke. For all groups, mice will be examined for nasal wash,

bronchoalveolar lavage fluid (BAL) and lung tissue to evaluate ECM degradation, bacteria colonization, airway, and lung inflammation.

### *Lung histology H&E staining*

Lungs were inflated with 10% neutral-buffered formalin, and fixed overnight at room temperature. Fixed tissues were dehydrated through graded ethanol, cleared in xylene, and embedded in paraffin. Sections (4–5 µm) were cut using a microtome, mounted on glass slides, and dried. Slides were deparaffinized, rehydrated, and stained with hematoxylin followed by eosin using standard protocols. After dehydration and clearing, sections were cover-slipped with mounting medium. Histological images were captured at 100× magnification using a light microscope. Slides were randomized, read blindly, and examined by a board certified veterinary anatomic pathologist (J.B.F) for tissue damage according to previously published scoring scheme to assess lung injury secondary to viral infection. Scores were broken down into distinct regions that included airway, vascular, and alveolar pathology in affected as previously published (2). The airway, alveolar, and vascular scores were then added together to obtain an overall lung injury score.

### *PE activity assay*

PE activity was measured using a fluorometric assay in black 96-well plates. Each well contained 70 µL reaction buffer (100 mM sodium phosphate, pH 7.5, supplemented with 1 mM freshly prepared DTT), 20 µL of sample or recombinant PE standard, and 10 µL of 1.0 mM AMC substrate (Suc-Gly-Pro-AMC; Bachem, Torrance, CA), for a final volume of 100 µL. Plates were incubated at 37 °C for 1 h, and fluorescence was recorded at 380 nm excitation and

460 nm emission using a microplate reader. PE activity was calculated from a standard curve generated with recombinant PE.

***MMP-9 and MPO ELISA assay***

MMP-9 and MPO protein levels were measured using mouse MMP-9 and MPO DuoSet ELISAs (DY6718 and DY3667, R&D Systems) following the manufacturer's instructions. Briefly, 96-well plates were coated overnight at room temperature (RT) with kit-provided capture antibody diluted in PBS. After washing and blocking, samples and standards were treated with kit-provided detection antibody, followed by the streptavidin-HRP method. Absorbance was measured at 450 nm in a microplate reader. Data were normalized to a standard curve.

***Quantification of PGP peptides by ESI-LC-MS/MS***

Proline-Glycine-Proline (PGP) was quantified using electrospray ionization-liquid chromatography-tandem mass spectrometry (ESI-LC-MS/MS) on an SCIEX 6500 QTRAP mass spectrometer (Sciex, Foster City, CA) coupled to a Shimadzu Prominence HPLC system with a refrigerated autosampler SIL-20A (Shimadzu Scientific Instruments, Columbia, MD). Chromatographic separation was performed on a Jupiter 4 µ Proteo column (80 Å, 150 × 2.1 mm; Phenomenex, Torrance, CA) maintained at 40 °C. The mobile phases consisted of water with 0.1% formic acid (A) and acetonitrile with 0.1% formic acid (B), delivered at 0.5 mL/min with a gradient from 5% to 100% B over 0.5–2.5 min, followed by column flushing with 100% mobile phase B. The injection volume was 30 µL, and the total run time was 5 min. Mass spectrometric detection was performed in positive electrospray ionization mode with an ion spray voltage of 5000 V, interface temperature of 600 °C, and gas settings of 45, 50, and 50 psi for curtain, GS1, and GS2, respectively. Multiple reaction monitoring (MRM) transitions were monitored at m/z

270→70 and 270→116 for PGP. Data acquisition and analysis were performed using Analyst software (version 1.7.2) and MultiQuant software (version 3.0) for respectively (SCIEX, Foster City, CA).

### *Statistical analysis*

Data conforming to normal distribution were presented as mean ± standard deviation. One-way ANOVA was performed with multiple comparison testing using Tukey's method and Mann–Whitney *U*-test was performed on data for comparison between two groups. GraphPad Prism version 10.0.2 (GraphPad Software; La Jolla, California) was used for all data analyses. $^{*}P < 0.05$ indicates statistical significance.

### *Study approval*

All experiments involving animals were conducted in accordance with the institutional guidelines set forth by the University of Alabama at Birmingham (UAB, Birmingham, AL USA). All animal protocols were submitted and approved by the IACUC of the University of Alabama at Birmingham (IACUC 22066) consistent with the Public Health Service policy on Humane Care and Use of Laboratory Animals (Office of Laboratory Animal Welfare).

### *Data availability*

Raw data underlying figures is available in Supporting data file and further information is available by contacting corresponding author.

**Supplemental Figure 1**

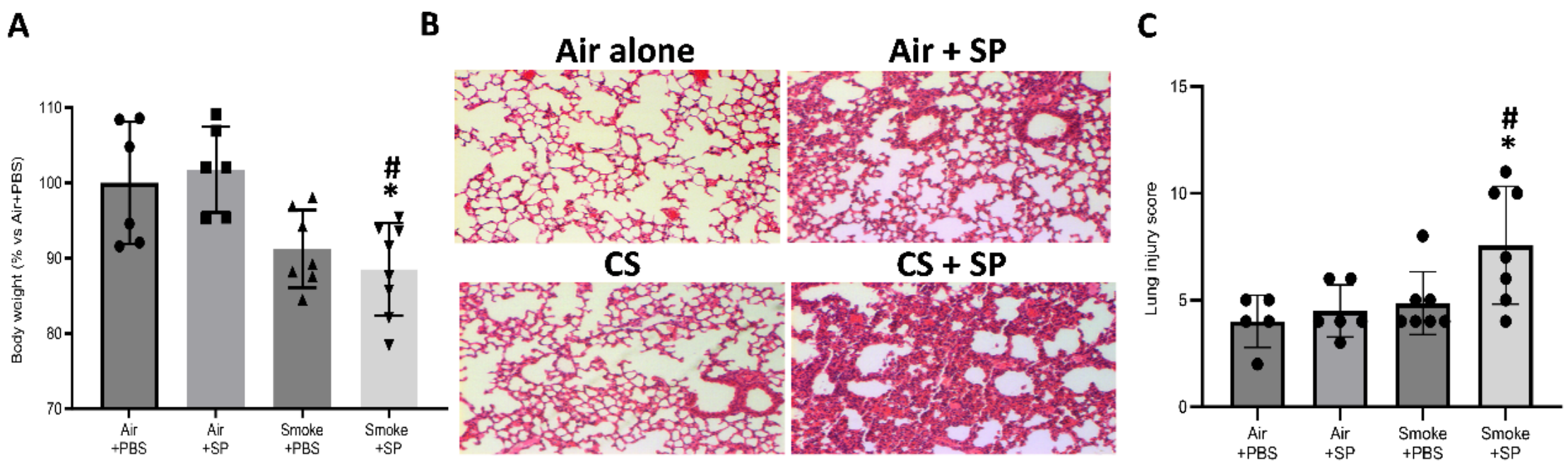


**Supplemental Figure 1. The lung inflammation in S. pneumoniae and cigarette smoke exposed mice:** Mice were exposed to cigarette smoke (CS) for 2 weeks and 2 x $10^6$ CFU S. pneumoniae (SP) was administered by intranasal (i.n.) two day before sample collection. (**A**) Body weight; (**B**) The lung Hematoxylin & Eosin (H&E, 100 x magnification) staining. (**C**) Lung injury score. Statistical analysis was performed using one way ANOVA with Tukey's multiple comparison post-test. #p<0.05 vs Air+PBS. *p<0.05 vs Air+SP. All values represent means ± SD.

**Reference**

1. *Kadioglu A, et al.* Sex-Based Differences in Susceptibility to Respiratory and Systemic Pneumococcal Disease in Mice. *J Infect Dis.* 2011;204:1971–1979.

2. Schultz, Suschak, J. J., Botta, D., Silva-Sanchez, A., King, R. G., Detchemendy, T. W., Lund, F. E.. et al. A single intranasal administration of AdCOVID protects against SARS-CoV-2 infection in the upper and lower respiratory tracts. *Human Vaccines & Immunotherapeutics*, 2022; 18(6): e2127292 -1-11

**Conflict of interest:** SWR, JL KRG, LV, JBF, LW, WES, JEB, AG, and XX have no conflicts of interest to report.

**Funding Support:**

This work is the result of NIH funding, in whole or in part, and is subject to the NIH Public Access Policy. Through acceptance of this federal funding, the NIH has been given a right to make the work publicly available in PubMed Central.

- National Institutes of Health (NIH) R35HL135710 (to JEB), R01HL162705 (to KRG)
- Veterans Administration (VA) Merit Review 101CX002751 (to AG) and VISN 7 RDA (to SWR)
